# Systematic review and meta-analysis of single-stage vs two-stage revision for periprosthetic joint infection: a call for a prospective randomized trial

**DOI:** 10.1186/s12891-024-07229-z

**Published:** 2024-02-19

**Authors:** Yong Zhao, Shaohua Fan, Zhangfu Wang, Xueli Yan, Hua Luo

**Affiliations:** 1 Department of Orthopedics, Shanghai Fengxian District Central Hospital, Shanghai, 201400 China; 2 Department of Orthopedics, Taizhou Hospital of Zhejiang Province affiliated to Wenzhou Medical University, Taizhou, 317000 Zhejiang China

**Keywords:** Periprosthetic joint infection, Single-stage, Two-stage, Reinfection, Reoperation

## Abstract

**Background:**

Periprosthetic joint infection (PJI) is a severe complication of joint arthroplasty that causes significant pain and economic loss. This study aimed to determine whether the current evidence supports single-stage revision for PJI based on reinfection and reoperation rates.

**Methods:**

We searched the PubMed, EBSCO, Medline, and Cochrane Library databases from inception to 30 May 2023 to identify studies that compared single-stage revision and two-stage revision for PJI. Data on reinfection and reoperation rates were pooled.

**Results:**

This meta-analysis included a total of 40 studies with 8711 patients. Overall, there was no significant difference between single- and two-stage revision regarding the postoperative reinfection rate and reoperation rate. Subgroup analysis by surgery period and different surgical sites revealed no difference between the two groups in the reinfection and reoperation rates.

**Conclusions:**

Based on the available evidence, our study did not identify a significant difference in reinfection and reoperation rates between single- and two-stage revision for PJI. Given the limitations in inclusion/exclusion criteria and the observed heterogeneity, we acknowledge the complexity of drawing strong conclusions. Therefore, we suggest that the choice between single- and two-stage revision should be carefully considered on an individual basis, taking into account patient-specific factors and further research developments.

**Supplementary Information:**

The online version contains supplementary material available at 10.1186/s12891-024-07229-z.

## Background

As a terminal means for treating osteoarthritis, joint arthroplasty can effectively reduce pain and improve quality of life. However, periprosthetic joint infection (PJI) is a severe complication of joint arthroplasty that causes significant pain and economic loss. It is expected that 10,000 patients with PJI will require revision each year by 2030 [1]. In recent years, improvements in surgical techniques and surgical conditions have led to a decline in the incidence of PJI from 1%–23% to 1%–2% [2]. With improvements in economic conditions and people's quality of life requirements, the total number of arthroplasty procedures have increased rapidly, and the number of PJIs has increased accordingly. Two-stage revision is considered the gold standard for treating PJI [3, 4]. However, the ideal time interval between surgical treatments, optimal antimicrobial agent, and duration of treatment remains controversial and the reported postoperative infection recurrence rate varies widely. In addition, some patients are in poor physical condition and may not be able to tolerate a second surgery. In recent years, the single-stage revision technique has received widespread attention and its application is increasing worldwide. Compared with two-stage revision, single-stage revision is more conducive to the functional recovery of the affected limb, reduces the occurrence of complications, reduces the overall treatment cost, reduces the surgical trauma, and improves patient satisfaction [5–7]. Moreover, several studies have reported comparable success with single-stage revision versus two-stage revision [6, 8, 9]. However, the evidence regarding single- and two-stage revision for PJI is inconsistent. This meta-analysis aimed to determine whether the reinfection and reoperation rates differ between the two treatment modalities and to ultimately reduce uncertainty in clinical decision-making for PJI treatment.

## Methods

According to the PRISMA (Preferred Reporting Items for Systematic Reviews and Meta-Analyses) statement, this meta-analysis was performed in agreement [10]. The protocol for this meta-analysis was registered on PROSPERO (Registration No: CRD 42022369943).

### Inclusion criteria

Study type: randomized controlled trial, cohort study, or retrospective study (Level I to III evidence). Study population: patients undergoing PJI. Intervention and control: single-stage in the treatment group, two-stage in the control group. Outcome index: clear reinfection or reoperation reported. Reinfection can be defined as the recurrence of clinical, serologic, or radiographic signs of infection during the follow-up period after the initial infection has been controlled. Reoperation can be defined as the patients need for further revision surgery.

### Exclusion criteria

Letters, case reports, reviews, animal trials, or republished studies; Studies lacking a control group; Patients with septic arthritis or tuberculous arthritis.

### Search strategy

Two of the authors (YZ and HL) performed the search in PubMed, EBSCO, Medline, and the Cochrane Central Register of Controlled Trials from the inception dates to May 30, 2023, using the keywords “(Two-stage or 2-stage or two stage or second-stage or double-stage) and (Single-stage or one-stage or 1-stage) and (arthroplasty or replacement) and (unhealed or infection or reoperate* or revise)”. No language restrictions were applied during the search.

### Study selection

Two researchers (YZ and ZFW) screened the retrieved literature strictly and individually against inclusion and exclusion criteria. If two researchers do not agree during the literature screening process, it will be left to the senior researcher (HL).

### Data collection process

Data on relevant outcome measures were extracted from the literature that met the inclusion criteria, including first author, year of publication, number of patients included, population characteristics (age, gender, comorbidities, etc), study design, PJI definition criteria, used, joint, surgical strategy, definition of failure (reoperation for infection, DAIR (debridement, antibiotic and implant retention), suppressive antibiotics), reason for reoperation other than infection and timming, follow-up by two researchers (SHF and HL) individually.

### Outcomes

The primary outcome was the incidence of reinfection. A secondary outcome was the incidence of reoperation.

### Assessment of risk of bias and quality of evidence

Two researchers (HL and SHF) independently assessed the quality of all included trials based on Cochrane risk-of-bias criteria [11]. The Newcastle–Ottawa scale (NOS) was used to evaluate the literature quality of the retrospective studies [12].

### Data synthesis

The Meta-analysis was performed using Stata (version 17; StataCorp, 2021) software. The heterogeneity was assessed by using the Q test and I^2^ value calculation. Suppose the heterogeneity was not present (*P*>0.1 and I^2^<50%), the data was combined with a fixed effect model. The random effects model was used if heterogeneity was present (*P*<0.1 or I^2^ >50%). The odds ratio (OR) and their associated 95% confidence interval (CI) were used to assess outcomes, and a *P* value less than 0.05 suggested that the difference was statistically significant.

### Subgroup analyses

We performed subgroup analyses for different surgical areas and periods of surgery.

### Sensitivity analyses

We performed a sensitivity analysis on a case-by-case exclusion basis using random effect models.

## Results

**Fig. 1:**
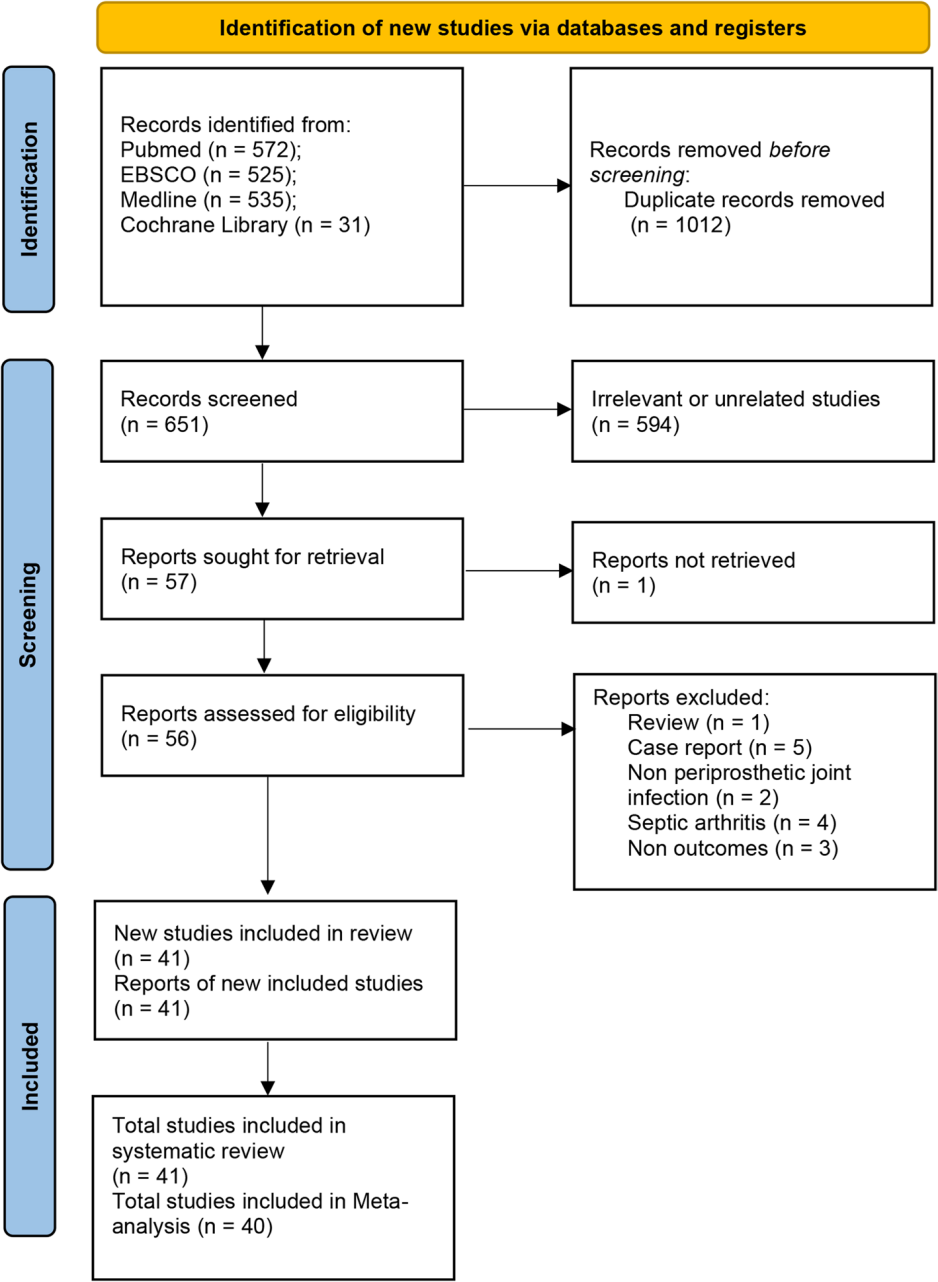
Flow diagram for search and selection of included studies

A total of 1663 documents were retrieved, 1012 duplicate documents were eliminated, the remaining 651 documents were read for abstracts and titles, 594 irrelevant documents were excluded, and 1 document failed to obtain the full text. The remaining 56 articles were read in full text. Fifteen studies were excluded, of which one review study, five case reports, three outcomes were no recurrence of infection, and six participants were without PJI. A total of 41 studies were included in the systematic review [5–9, 13–48], of which one study was excluded from the meta-analysis as the reinfection or reoperation outcomes could not be extracted [26]. A total of 40 articles were included in the meta-analysis (Fig. 1). The characteristics of the included studies are detailed in Table 1.

**Table 1:** Characteristics of included studies

Study	No. of subjects		Country	Period of surgery	Participants	Design	Minimal follow-up	Part of component removed	Failure /reinfection definition	Statistical analysis	Outcomes	Age		NOS Scores	PJI definition	Criteria for select	
	Single-stage	Two-stage										Single-stage	Two-stage			Single-stage	Two-stage
Baker 2013 [5]	33	89	UK	2008-2010	Infected TKA	Retrospective cohort	6 (6-12) M	NA	NA	Yes	Reoperation, knee score, satisfaction	69.4 ± 10.7	70.3 ± 8.9	8	NA	NA	NA
Bauer 2006 [13]	30	77	France	NA	Infected TKA	Retrospective cohort	2 Y	NA	NA	Yes	Reinfection rate, knee score	71.8	68.3	8	Multiple sample germs	NA	NA
Betsch 2008 [14]	4	51	Switzerland	1995-2004	PJI (Hip and Knee)	Retrospective cohort	24 M	Infected prosthesis	Yes	Yes	Failure rate	67.1 ± 11.7		9	Sinus tract, positive cultures, tissue neutrophils, or pus hint infection	NA	NA
Castellani 2017 [15]	35	75	Canada	2000-2013	PJI (Hip and Knee)	Retrospective cohort	12 M	Components	Yes	Yes	Failure rate	68	63	9	Visible infection signs, positive cultures, or pre-op sinus tract	NA	NA
Chalmers 2020 [16]	1	4	USA	2009-2017	PJI following UKA	Retrospective cohort	1 (1-9) Y	All components	Yes	Yes	Reinfection, reoperation	51-87		9	MSIS	NA	NA
Choi 2013 [17]	17	44	USA	1999-2009	Infected THA	Retrospective cohort	12 (12-132) M	All components	Yes	Yes	Reinfection, hip score	NA	NA	9	MSIS	Based on patient specifics and surgical conditions	
Crego 2019 [18]	20	45	Germany	NA	PJI (Knee)	Retrospective cohort	NA	NA	NA	Yes	Reinfection, hip score	NA	NA	9	NA	NA	NA
Cristea 2007 [19]	10	25	Romania	1990-2004	PJI (Hip and Knee)	Retrospective cohort	NA	NA	NA	Yes	Reinfection	NA	N	6	Positive cultures	NA	NA
Cury 2015 [20]	6	7	Brazil	2008-2010	PJI after TKA	Retrospective cohort	NA	NA	Yes	Yes	Reinfection, life scores	NA	NA	6	NA	Good skin condition, no major health issues, and antibiotic-sensitive	Poor skin and tissue conditions
Engesater 2011 [21]	501	283	Norway	1987-2009	Infected THA	Retrospective cohort	NA	All or partial components	NA	Yes	Reoperation	71.5	71	8	NA	NA	NA
Gao 2008 [22]	10	5	China	1999-2005	Infected THAs	Retrospective cohort	12 (12-31) M	All components	NA	Yes	Reinfection, reoperation, hip score	54-71		6	NA	NA	NA
Haddad 2015 [23]	28	74	UK	2004-2009	Chronic infected TKA	Retrospective cohort	3 (3-9) Y	All components	Yes	Yes	Reinfection, reoperation, knee score	63 (48-87)	68 (45-85)	7	Same microorganism in 3+ surgical samples from different sites.	(1) minimal bone or soft tissue loss, (2) non-immunosuppressed patients without ongoing sepsis or chronic diseases, (3) isolated low-virulent pre-op organism sensitive to antibiotics. Excluded polymicrobial or multi-resistant infections like MRSA/MRSE. Decision after discussing with microbiologists.	Contraindications present in single-stage
Hope 1989 [24]	72	19	UK	1976-1987	Coagulase-negative staphylococci infected THA	Retrospective cohort	2 (2-121) M	All components	NA	Yes	Failure rate	64 (30-85)	65 (41-81)	8	Diagnosed clinically, hematologically, through imaging, and samples from joint and prosthetic tissues	NA	NA
Jacquot 2015 [25]	5	14	France	1996-2011	Infected RSA	Retrospective cohort	12 (12-137) M	NA	NA	Yes	Reinfection, complication rate	71 (55-83)		7	MSIS	By the surgeon, considering the patient’s age and comorbidities, surgical history, infection characteristics, and bone or soft tissue defects.	
Kheir 2017 [27]	11	43	USA	1991-2014	Enterococcal PJI (Knee and Hip)	Retrospective cohort	12 M	NA	Yes	Yes	Failure rate	66.3 (39-85)		9	MSIS	NA	NA
Klemt 2021 [9]	44	88	USA	2015-2018	Chronic PJI (Knee)	Retrospective cohort	NA	NA	NA	Yes	Reinfection, reoperation, readmission, patient-reported outcome measures	64.9 ± 9.2	65.4 ± 8.6	8	MSIS	Microorganism with low virulence, patient in good health without immunocompromising systemic conditions, absence of any septic focus, implant loosening, limited soft-tissue defect or condition that impedes direct closure of wound after revision surgery	Major tissue or bone loss, implant looseness, reinfection, resistant/unidentified germs
Klouche 2012 [28]	38	46	France	2002-2006	PJI (Hip)	Prospective non-randomised study	24 (24-68) M	All components	Yes	Yes	Reinfection	63.60 ± 14.8	66.87 ± 12.1	7	Positive cultures	Surgeon knew germ pre-exchange, deemed bone loss minor pre-op and during surgery post-component removal	No pre-op microbe diagnosis, major bone loss
Laffer 2006 [29]	2	13	Switzerland	1988-2003	PJI after TKA	Retrospective cohort	2 (2-193) M	NA	Yes	Yes	Success rate	70.1 (43.5–90.1)		8	Sinus tract to joint or two of: positive tissue/fluid culture, high neutrophils, high leukocytes, clinical signs, or radiological infection signs	NA	NA
Larsson 2018 [30]	9	46	Switzerland	2008-2012	PJI (Hip)	Retrospective cohort	12 (12-60) M	NA	Yes	Yes	Success rate	67 (31-90)		8	Consensus Meeting on Periprosthetic Joint Infection	NA	NA
Lecuire 1999 [31]	16	41	French	NA	Infected THA	Retrospective cohort	6.6 Y (mean)	NA	NA	NA	Reinfection, hip score	NA	NA	6	NA	NA	NA
Lemmens 2021 [32]	1	16	Belgium	2004-2018	Infected primary or revision RSA	Retrospective cohort	24 (24-132) M	NA	Yes	NA	Reinfection, functional outcome	66.8 (44-81)		8	International Consensus Meeting on Orthopedic Infections	Personalized based on bone health, age, overall health, expectations, and compliance, chosen at the surgeon's discretion	
Lenguerrand 2022 [7]	489	2377	International multicenter	2003-2014	Infected primary knee arthroplasty	Retrospective cohort	NA	NA	Yes	Yes	Reoperation	68 ± 10	69 ± 9	9	NA	NA	NA
Leta 2019 [33]	72	243	Norway	1994-2016	PJI after primary TKA	Retrospective cohort	1 Y	NA	NA	Yes	Reinfection, reoperation, survival rate, mortality rate	69 ± 9.5	69 ± 9.7	9	Based on the assessment of PJI and the clinical picture	NA	NA
Li 2017 [34]	22	105	China	2003-2014	Infected revision TKA	Retrospective cohort	12 (12-158) M	Infected prostheses	Yes	Yes	Reinfection, complication	64.4 ± 9.5		9	MSIS	Confirmed effective antibiotic treatment	Widespread infection symptoms with unclear cultures or resistant organisms or sinus tracts detected beforehand
Mahieu 2019 [35]	15	17	France	2010-2012	Streptococcal PJI (Knee and Hip)	Retrospective cohort	24 M	NA	NA	Yes	Reinfection, reoperation	77 (69–83)		7	IDSA	Based on center expertise, with no bone reconstruction needed, healthy soft tissue, and specific microorganism identification	All other situations
Massin 2016 [36]	108	177	France	2005-2010	Infected TKA	Retrospective cohort	2 Y	NA	NA	Yes	Reinfection, reoperation, knee score,	71 (63–76)	67 (59–73)	8	Sinus tract to joint or two of: positive tissue/fluid culture, high neutrophils, high leukocytes, clinical signs, or radiological infection signs	NA	NA
Matar 2021 [8]	82	210	UK	2003-2018	Chronic PJI (Knee)	Retrospective cohort	2 (2-17.6) Y	NA	Yes	Yes	Success rate, survivorship rate	71.8 ± 9.8	70.5 ± 10.2	8	MSIS	Single organism infection, known sensitivities, healthy immune system, intact soft tissues, and no systemic sepsis or draining sinus indicated suitability	NA
Ribes 2019 [6]	21	41	France	2009-2014	Chronic infected TKA	Retrospective cohort	1 Y	NA	Yes	Yes	Reinfection, knee score,	72.6 ± 9.2	69.5 ± 9.1	9	IDSA	Two-stage replacement was preferred when: unknown cause; resistant bacteria to effective antibiotics; skin or fistula issues hindering closure; significant pus risking new prosthesis contamination; substantial bone defects needing grafting.	
Ritter 2010 [37]	8	68	Indiana	1969-2004	PJI (Knee and Hip)	Retrospective cohort	1 Y	NA	Yes	Yes	Success rate	65.4 ± 12.2		9	NA	NA	NA
Siddiqi 2019 [38]	57	137	USA	2012-2017	Chronic PJI after primary TKA	Retrospective cohort	2 Y	NA	Yes	Yes	Reinfection, reimplantation, reoperation rates, success rate	NA	NA	9	MSIS	NA	NA
Sotiriou 2022 [39]	6	42	Sweden	2002-2016	PJI after THA	Retrospective cohort	2 Y	NA	Yes	Yes	Reinfection, reoperation, operating time, hospital stay, blood loss, hip score	73 ± 11.8	68 ± 10.8	9	History, physical exam, sedimentation rate, C-reactive protein, and bacterial cultures (joint fluid aspiration or biopsy)	NA	NA
Stone 2017 [40]	60	29	USA	2004-2012	PJI (Shoulder)	Retrospective cohort	12 (12-105) M	All or partial components	Yes	Yes	Reinfection, reoperation, complication, functional outcome	69 (30-92)	65 (27-73)	9	Combining previous infection history, physical signs (like skin changes, swelling, draining sinus), lab tests (white cell count, sedimentation rate, C-reactive protein), and positive intraoperative findings (like pus, specific cell count in frozen sections, and cultures).	NA	NA
Svensson 2019 [41]	404	1250	Sweden	1979-2015	Infected primary THA	Retrospective cohort	8.6 Y (mean)	All components	NA	Yes	Reinfection, reoperation, survival rate,aseptic loosening	70 ± 10	68 ± 10	8	NA	NA	NA
Tirumala 2021 [42]	46	92	USA	2014-2018	Chronic PJI (Hip)	Retrospective cohort	17.8 M (mean)	All components	NA	Yes	Reinfection, reooperation, mortality rate, functional outcome	68.88 ± 9.47	68.17 ± 8.28	9	MSIS and ICM	A mild microorganism infection in a healthy patient without immune system issues, with no visible infection site or implant issues	Severe soft tissue or bone damage, implant looseness, reinfection, resistant or unknown organisms
Tuecking 2021 [43]	15	48	Germany	2013-2019	Late-onset PJI (Knee)	Retrospective cohort	18 (18-92) M	NA	Yes	Yes	Reinfection, reoperation, implant survival	65.0 ± 10.2	69.3 ± 11.1	9	EBJIS	NA	NA
Van den Kieboom 2021 [44]	30	75	USA	2010-2018	Chronic culture-negative PJI (Knee and Hip)	Retrospective cohort	2.5 (2.5-22.9) Y	NA	Yes	Yes	Reinfection, reoperation, amputation, readmission, mortality, hospital stay	67.9 ± 10.6	65.0 ± 11.0	9	MSIS	NA	NA
Van Dijk 2022 [45]	21	107	Netherlands	2010-2017	PJI (Knee and Hip)	Retrospective cohort	4 Y	Infected prosthesis	Yes	Yes	Reinfection, survival rate	72 (55-92)	70 (44–92)	9	MSIS	Good for infections with effective antibiotics, not for severe systemic infections or when the organism isn't known before surgery or if there's significant soft tissue involvement needing flap coverage	NA
Wolf 2014 [46]	37	55	Austria	1985-2004	Infected THA	Retrospective cohort	2 Y	NA	NA	Yes	Reinfection	67	60.4	9	Signs include high white blood cells, increased CRP, redness, swelling, warmth, fluid aspiration, and positive microbiology	NA	NA
Wouthuyzen-Bakker 2019 [47]	20	78	International multicenter	2005-2015	Late acute PJI (Hip and Knee)	Retrospective cohort	10 (10-55) M	All components	Yes	Yes	Failure rate	NA	NA	9	MSIS	NA	NA
Xu 2022 [48]	13	36	China	2012-2017	PJI (Hip and Knee)	Retrospective case-control	2 Y	NA	Yes	Yes	Success rate, complication, remission rate	63.6		9	NA	NA	NA

*PJI* periprosthetic joint infection, *TKA* total knee arthroplasty, *THA* total hip arthroplasty, *RSA* Reversed shoulder arthroplasty, *NOS* Newcastle–Ottawa scale, *M* month, *Y* year, *NA* Not applicable, *MSIS* Musculoskeletal Infection Society, *IDSA* Infectious Diseases Society of America, *ICM* International Consensus Meeting, *EBJIS* European Bone and Joint Infection Society

A total of 41 retrospective studies were included in our systematic review. We used the NOS to assess the methodological quality and risk of bias. The quality scores were 6 to 9, indicating an overall low risk of bias (Table 1).

### Reinfection

**Fig. 2:**
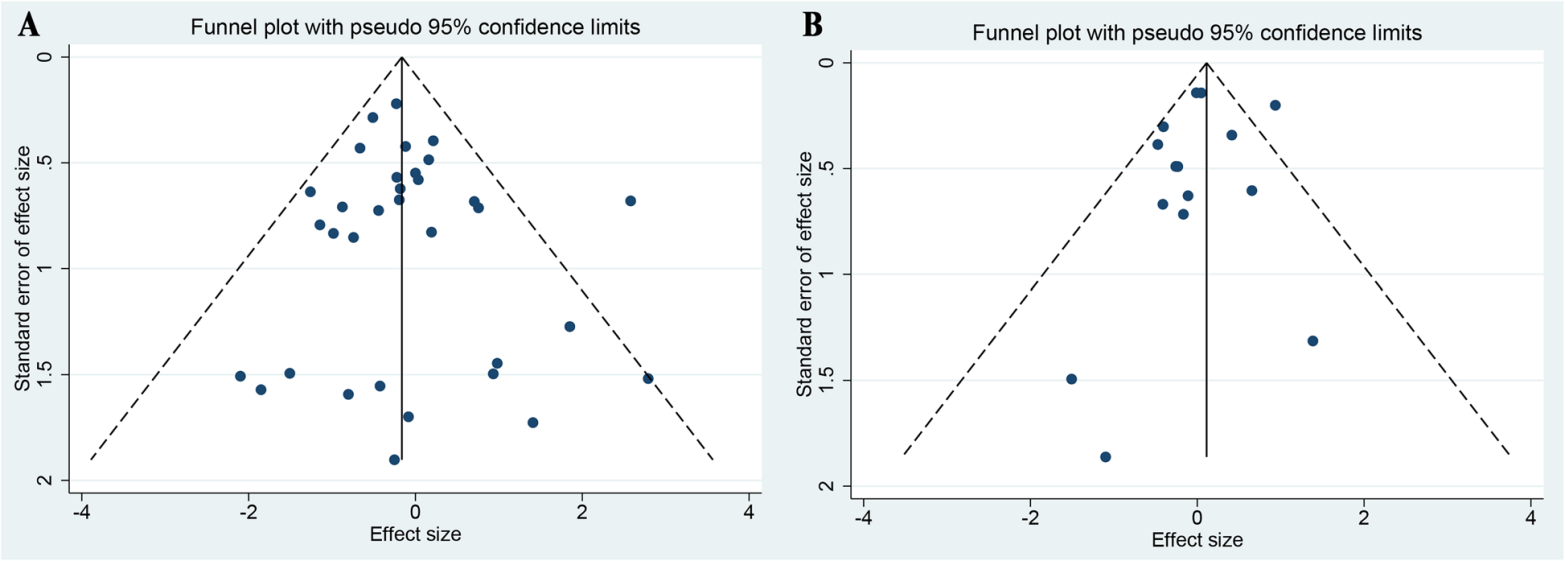
Funnel plot of the included studies in this meta-analysis for the incidence of reinfection (**A**) and reoperation (**B**)

A total of 37 studies reported the recurrence of infection [6, 8, 9, 13–20, 22–25, 27–36, 38–49]. Van den Kieboom et al. [44] included both superficial and deep infections. We did not exclude superficial infections as these may result in deep infections. Among the cohort evaluated by Larsson et al., [30] we excluded one patient in the single-stage group who experienced treatment failure because the appropriate criteria were not met. There was mild heterogeneity between studies (I^2^=24.3%, *P*=0.106), and a fixed-effect model was used. There was no difference in the reinfection rate after single- versus two-stage revision for PJI (OR: 0.88; 95% CI: 0.73–1.07; *P*=0.209; Fig. S1A). As different surgical sites and surgery periods may have been a source of heterogeneity, subgroup analyses were performed. There was no difference in the reinfection rate between the single- and two-stage groups among the subgroups with PJI of the hip (OR: 1.35; 95% CI: 0.66–2.76; *P*=0.410; I^2^=53.5%; Fig. S1B), knee (OR: 0.76; 95% CI: 0.58–1.00; *P*=0.052; I^2^=0%; Fig. S1B), or shoulder (OR: 0.55; 95% CI: 0.16–1.88; *P*=0.338; I^2^=0%; Fig. S1B). Subgroup analysis based on the surgery period showed no significant difference in the reinfection rate between the single- and two-stage groups that underwent surgery after 2005 (OR: 0.79; 95% CI: 0.58–1.08; *P*=0.142; I^2^=0%; Fig. 2C), during both surgery periods (OR: 0.78; 95% CI: 0.58–1.07; *P*=0.125; I^2^=0%; Fig. S1C), or before 2005 (OR: 2.69; 95% CI: 0.58–12.37; *P*=0.204; I^2^=60.6 %; Fig. S1C).

### Reoperation

A total of 18 studies reported the number of reoperations [5, 7–9, 16, 21–23, 33, 35, 36, 38–44]. There was no significant difference in the reoperation rate between the single- and two-stage groups (OR: 1.04; 95% CI: 0.79–1.37; *P*=0.792; I^2^=52.2 %; Fig. S2A). Considering the heterogeneity of the results, subgroup analyses were performed for different surgical sites and surgery periods. Subgroup analyses showed no difference in the reoperation rate after single-stage revision versus two-stage revision for PJI of the hip (OR: 1.49; 95% CI: 0.77–2.89; *P*=0.239; I^2^=76.6%; Fig. S2B), knee (OR: 0.93; 95% CI: 0.74–1.16; *P*=0.509; I^2^=2.6%; Fig. S2B), or shoulder (OR: 1.10; 95% CI: 0.31–3.75; *P*=0.880; Fig. S2B). Subgroup analysis based on the surgery period showed no difference in the reoperation rate between the single- and two-stage groups that underwent surgery after 2005 (OR: 0.77; 95% CI: 0.55–1.08; *P*=0.129; I^2^=0%; Fig. S2C) or during both surgery periods (OR: 1.23 95% CI: 0.82–1.83; *P*=0.316; I^2^=69.2%; Fig. S2C). Only one study that reported reoperation data was performed before 2005, and statistical calculations could not be performed because the number of events in both groups was 0.

### Sensitivity analysis

A sensitivity analysis of the included studies was performed on a case-by-case exclusion basis. The remaining studies were combined using the OR values if any study was excluded. No individual study had a significant impact on the results (Fig. S3A and B).

### Risk of bias

As shown in Fig. 2, the funnel plots showed some asymmetry, but the Harbord test showed no evidence of publication bias regarding reinfection (*P*=0.537) and reoperation (*P*=0.322).

## Discussion

Several systematic reviews and meta-analyses have explored reinfection rates after single- or two-stage revision, but the two treatment protocols were not compared and the studies were limited to a single surgical site analysis [49–56]. Nagra et al. [57] published a meta-analysis of the two treatment options in 2016, but included only five retrospective studies comparing 796 patients with PJI of the knee. Since the publication of the meta-analysis by Nagra et al., [57] there has been a significant increase in studies reporting reinfection rates after single-stage revision for PJI of the knee. Therefore, we searched the literature for relevant studies and included studies evaluating the treatment of knee, hip, and shoulder PJI to determine whether the reinfection and reoperation rates differed between the single- and two-stage revision groups.

Our study found no difference in the reinfection and reoperation rates between the single-and two-stage groups. The decision whether to perform a single or two-stage revision is made at the discretion of the surgeon after considering all the details of the patient and the surgical site; therefore, there was some allocation bias that led to this result. In addition, for patients with hip and shoulder PJI, only part of the prosthesis may be revised [21, 40], leading to incomplete debridement and an increased reinfection rate after single-stage revision. The reoperation rate did not differ between the single-stage and two-stage groups for different surgical sites. As the data collected for the current analysis spanned a long period from 1969 to 2019, which may constitute a potential confounding source for our analysis, we performed subgroup analyses of surgery periods. These subgroup analyses showed no significant differences in the rates of reinfection and reoperation between the two groups. Svensson et al. [41] defined reinfection as the need for reoperation due to reinfection. However, some patients may have had secondary infections that resolved with medication and did not require a second revision surgery. This may have led to increased reporting bias.

Although two-stage revision has traditionally been considered the gold standard for treating PJI [58], it significantly reduces patient activity time to a total of approximately twice as long as single-stage revision. Removal of a well-immobilized prosthesis may also lead to degeneration of bone stock and perioperative fractures [21]. Single-stage revision offers advantages such as a similar failure rate as a two-stage replacement, reduced hospitalization and costs, and improved cost-effectiveness [58, 59]. Our findings suggest that there was no difference between single- and two-stage revision in the rates of reinfection and reoperation. Studies have found that the risk factors for failure of single-stage revision may be related to prior joint infection with *Enterococcus* or *Streptococcus* species [60], so it may be interesting to compare the outcomes of single-stage revision to treat PJI caused by these two bacterial species. Moreover, different studies have used different surgical procedures and methods for the criteria for single- versus two-stage revision, and there is no regulation of the use of antibiotics. The present study focused on whether single-stage revision can achieve the same treatment effect as two-stage revision while reducing the surgery time, pain, and cost. Therefore, more comparisons of antibiotics and optimization of surgical procedures need to be performed to provide a basis for formulating relevant guidelines.

### Strengths

This is the first comprehensive comparison of the efficacy of single- and two-stage revision for PJI. This meta-analysis pooled 40 published studies involving 8711 patients with PJI, which may improve the statistical power of the data analysis and thus provide more reliable estimates. Sources of heterogeneity were analyzed, and subgroup analyses were performed for different surgical sites and periods of surgery. Our results showed that the success rate of single-stage revision was comparable to that of two-stage revision, challenging the assumption that two-stage revision is the gold standard for PJI. Clinicians are encouraged to consider single-stage revision for eligible patients with PJI. Compared with studies within a single country, our study pooled relative data from multiple countries worldwide, enhancing the universal applicability of the findings. Based on the Harbord tests and funnel plots, there was no significant publication bias in the included studies. Therefore, the results based on the available evidence are compelling.

### Limitations

This study has several limitations. First, the most significant limitation of our article is that the included studies were all non-randomized controlled studies. The allocation of patients was not based on randomization but rather on the surgeons' experience, resulting in a preference for two-stage revision in patients with contraindications to single-stage revision or those with severe joint infection [9, 28], leading to allocation bias. Therefore, the confidence of the results needs to be further confirmed by randomized controlled trials. Second, the definition of reinfection after revision differed between studies. Castellani et al. [15] defined the outcome as a failure without stating the rates of reinfection or revision. Thus, we could only judge whether patients had reinfection based on the description of the definition in the complete text, and discussed each patient to decide whether to include them in the group with reinfection, which may have deviated from the authors’ original definition [15]. Third, Kheir, [27] Mahieu et al., [35] and Van den Kieboom et al. [44] studied patients with specific bacterial infections or those with negative bacterial cultures, which increased the bias of the results. Fourth, the present review included studies with follow-up periods ranging from 6 months to 22 years. Some studies had a very long follow-up, and the reason for reoperation was independent of the surgical modalities; in other studies, the follow-up time needed to be longer, resulting in missing outcome measures. Fifth, when we performed statistical calculations, we did not adjust the original data in accordance with confounding factors but simply combined the original data statistically, which increased the bias of the article. Sixth, in the studies we included, both partial and complete implant removal were incorporated, to some extent, increasing the heterogeneity of the article.

## Conclusions

To our knowledge, this is the first meta-analysis to summarize the current evidence about the differences between single- and two-stage revision in treating PJI. We found that there was no difference between single- and two-stage revision in the reinfection and reoperation rates. Recognizing constraints in our inclusion/exclusion criteria and the observed diversity, we acknowledge the challenge of making definitive conclusions. Hence, we recommend a thoughtful, case-by-case consideration of the choice between single- and two-stage revision, considering patient-specific factors and staying attuned to ongoing research advancements.

### Supplementary Information

**Additional file 1.**

**Additional file 2: Figure S1.** Reinfection rate in included studies (**A**) Subgroup analysis of the reinfection according to different surgical sites (**B**) and surgery periods (**C**)

**Additional file 2: Figure S2.** Reoperation rate in included studies (**A**) Subgroup analysis of the reoperation according to different surgical sites (**B**) and surgery periods (**C**)

**Additional file 2: Figure S3.** The result of sensitivity analysis of reinfection (**A**) and reoperation (**B**)

## Data Availability

The datasets used and/or analyzed during the current study are available from the corresponding author on reasonable request.

## Abbreviations

PJI: Periprosthetic joint infection
OR: odds ratio
CI: confidence interval
