# Supplementary figures and images for: Systematic review and meta-analysis of single-stage vs two-stage revision for periprosthetic joint infection: a call for a prospective randomized trial
Source: BMC Musculoskelet Disord. 2024 Feb 19;25:153. doi: 10.1186/s12891-024-07229-z (PMC10875807; doi:10.1186/s12891-024-07229-z)

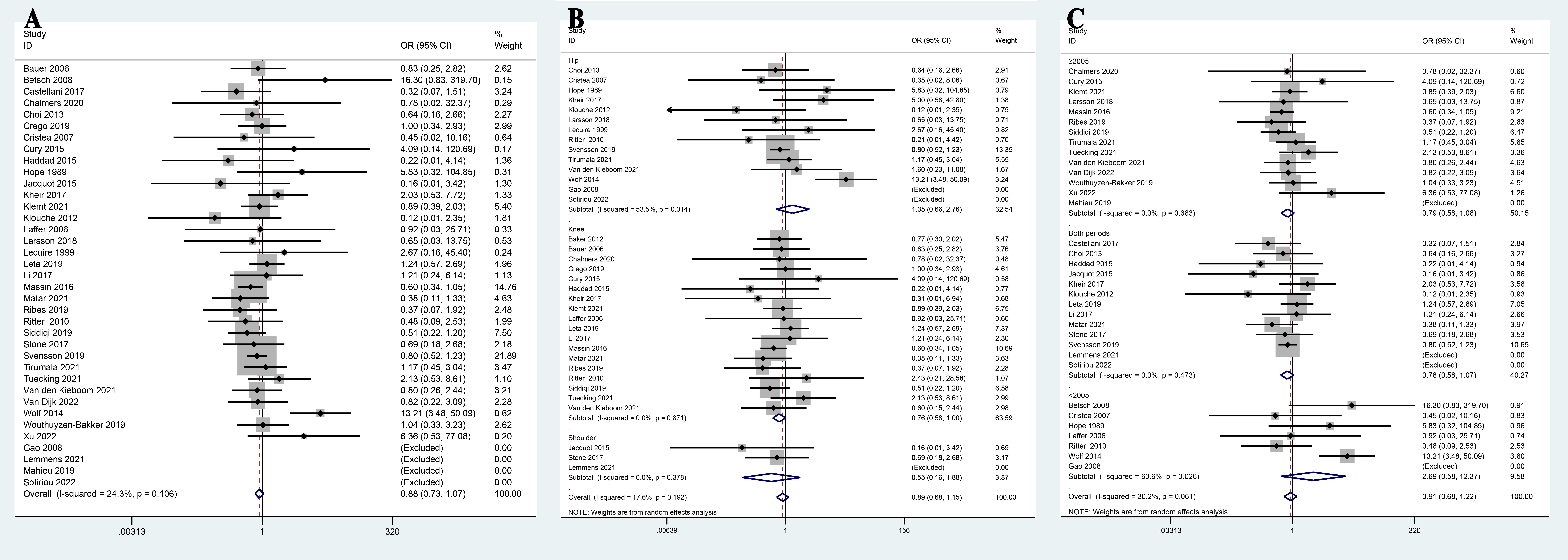

Supplement: Supplementary file 2 — Additional file 2: Figure S1. Reinfection rate in included studies (A) Subgroup analysis of the reinfection according to different surgical sites (B) and surgery periods (C) [file 12891_2024_7229_MOESM2_ESM.tif]

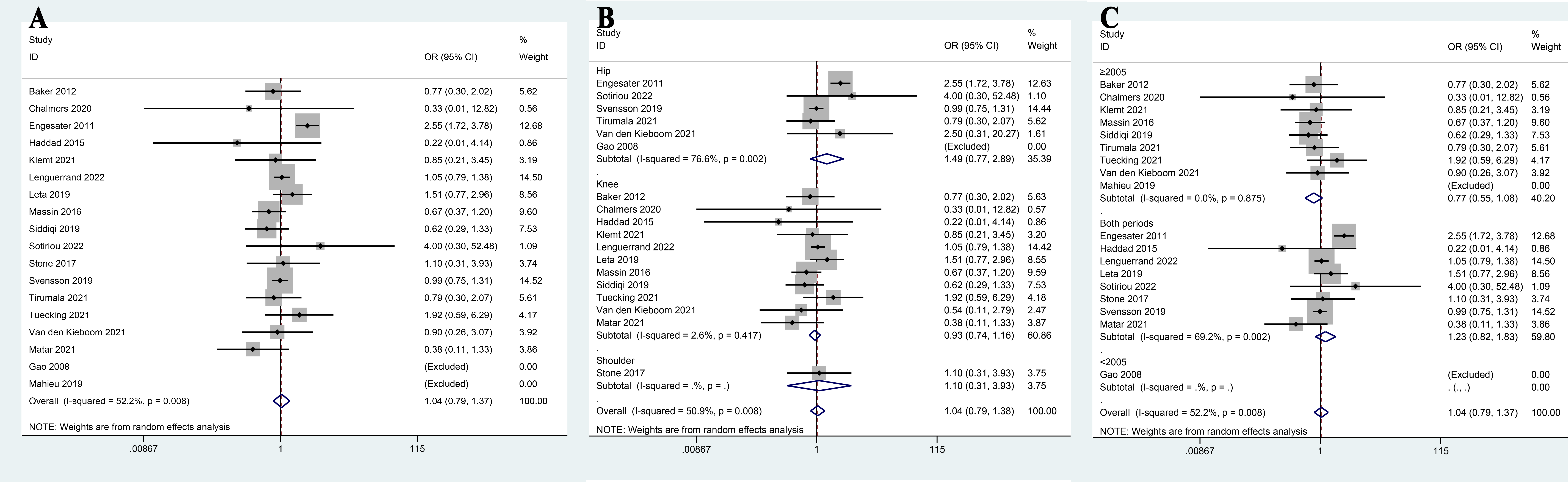

Supplement: Supplementary file 3 — Additional file 2: Figure S2. Reoperation rate in included studies (A) Subgroup analysis of the reoperation according to different surgical sites (B) and surgery periods (C) [file 12891_2024_7229_MOESM3_ESM.tif]

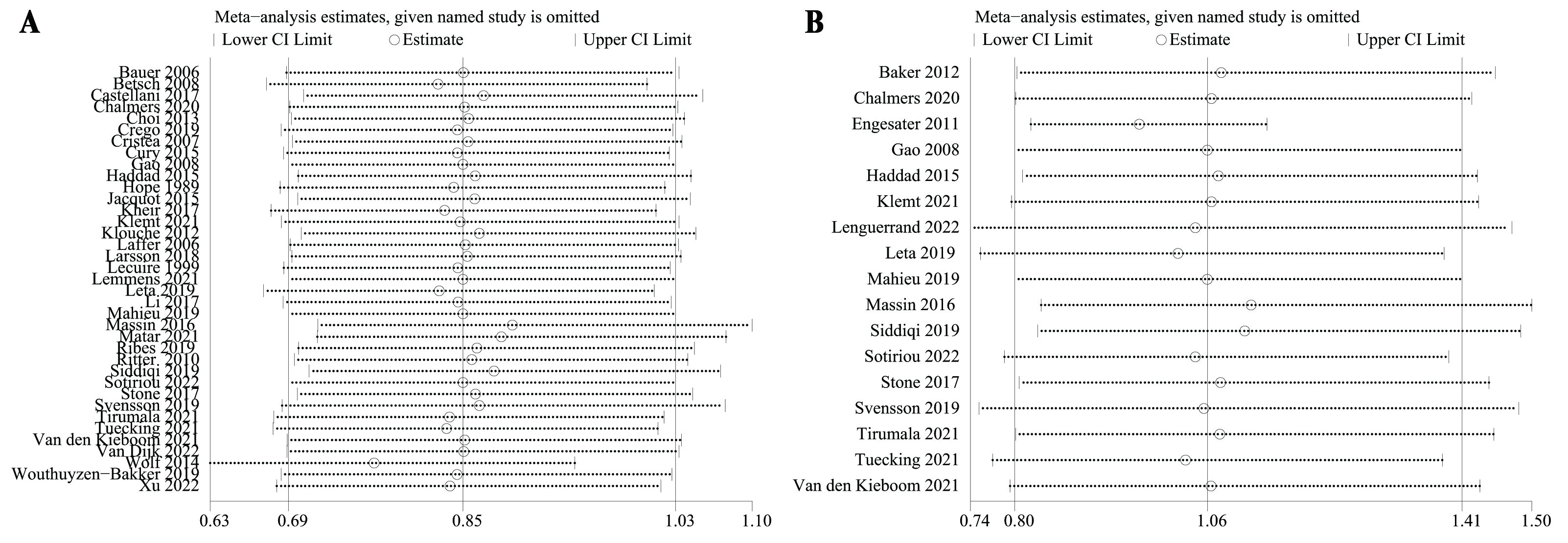

Supplement: Supplementary file 4 — Additional file 2: Figure S3. The result of sensitivity analysis of reinfection (A) and reoperation (B) [file 12891_2024_7229_MOESM4_ESM.tif]
